# Lower limb preservation in pediatric trauma: a case study of Gustilo grade IIIC fracture in a 7-year-old

**DOI:** 10.1093/jscr/rjae016

**Published:** 2024-01-24

**Authors:** Jamal Ataya, Rawan Daaboul, Hassan Alhomsi, Hassan Issa, Ahmad Elewee

**Affiliations:** Faculty of Medicine, University of Aleppo, Aleppo, Syria; Faculty of Medicine, AlBaath University, Homs, Syria; Faculty of Medicine, Damascus University, Damascus, Syria; Department of Orthopedic Surgery, Damascus Hospital, Damascus, Syria; Department of Orthopedic Surgery, Damascus Hospital, Damascus, Syria

**Keywords:** limb salvage, crush injury, Gustilo grade IIIC, internal fixation, case report

## Abstract

This case report describes the intricate aspects of managing pediatric lower limb trauma. A 7-year-old patient had a severe compound fracture and significant soft tissue damage in the left lower limb, classified as Gustilo Grade IIIC. This necessitated the use of scoring systems such as the Mangled Extremity Severity Score and limb salvage index to assess the likelihood of limb preservation. Despite these high amputation risk indicators, a multidisciplinary approach has led to limb salvage surgery with internal fixation. Detailed postoperative monitoring revealed progressive recovery culminating in restored sensation, bone healing, and functional recovery. The discussion emphasizes the difficulties in deciding between limb salvage and amputation, stressing the importance of tailored care and cautious scoring system interpretation in pediatric cases. This conclusion advocates the prioritization of limb salvage in children owing to their unique healing capabilities while highlighting the need for further research to refine treatment protocols for pediatric lower limb trauma.

## Introduction

Orthopedic and plastic surgeons often face challenges with open lower-extremity fractures caused by vascular trauma, resulting in severe hemorrhage or limb-threatening ischemia [1]. The primary objective was to establish bony union and adequate soft tissue coverage around the injured bone and hardware for optimal functional recovery. This complexity increases significantly in Gustilo Grade IIIB and IIIC fractures, where compromised nativevasculature adds further complexity [2].

Predictive models are used to assess the likelihood of lower-limb amputation in pediatric trauma cases. These models enable comprehensive evaluation of the surgical interventions necessary for successful limb salvage. Various scoring systems, such as the Mangled Extremity Severity Score (MESS) and limb salvage index (LSI), provide estimations of the probability of limb preservation [3, 4]. Prompt diagnosis and surgical intervention are crucial for reducing ischemic duration and amputation rates, highlighting the importance of a multidisciplinary team approach. Severe local infections can negatively impact overall outcomes, particularly in cases involving major soft tissue trauma or open fractures [5, 6].

This report describes a pediatric patient experienced a traumatic transection of the posterior tibial artery, an open fracture from the left tibia to the talus, and a concurrent subtalar and ankle dislocation. Treatment with internal fixation resulted in successful leg revascularization and bone healing.

## Case presentation

**Figure 1:**
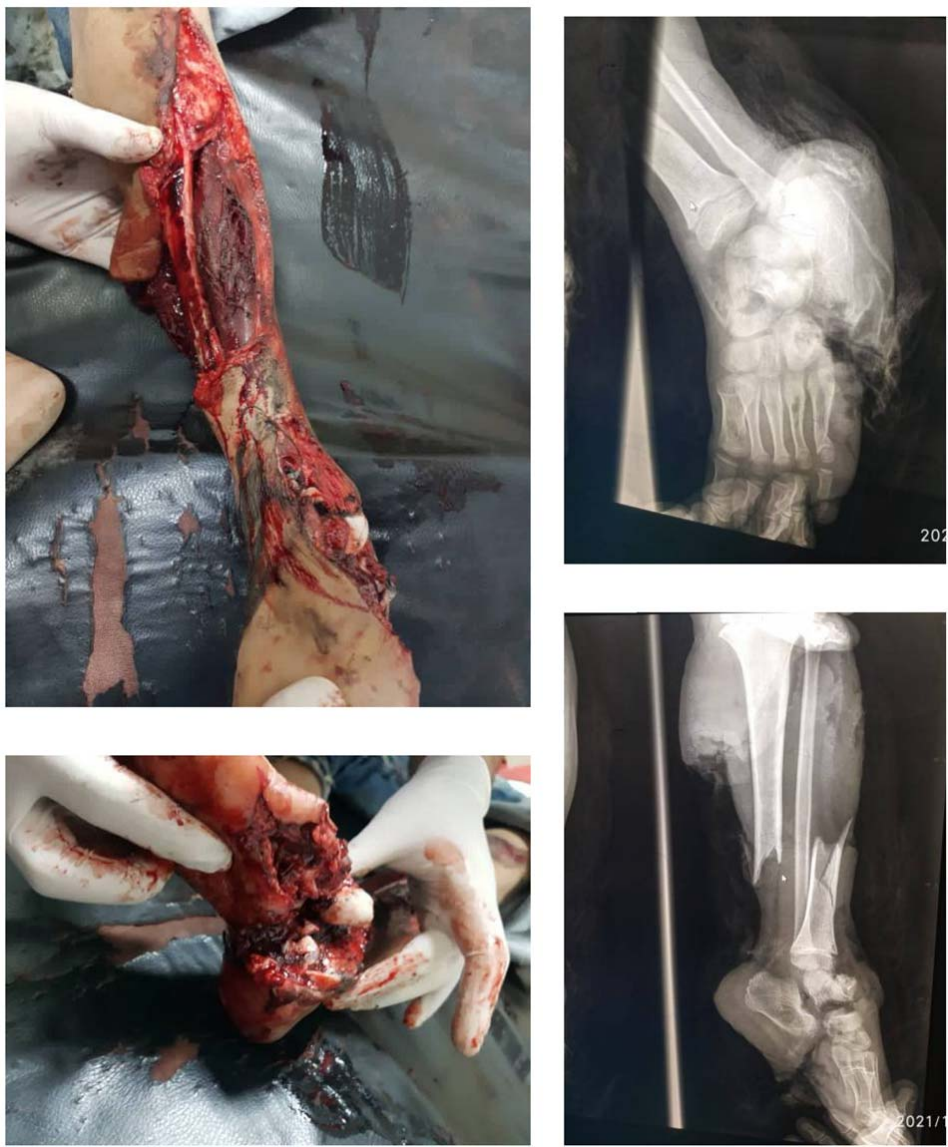
Displays the fractured image alongside the X-ray images taken prior to the intervention.

A 7-year-old boy experienced severe trauma to his left lower limb in a vehicular accident. At the emergency department, a comprehensive examination revealed a complex injury profile, including severe open fracture, dislocation of the subtalar joint and ankle, comminuted fracture of the cuboid with significant soft tissue loss, periosteal stripping, severe contamination, and lacerations in the posterior calf muscles. The limb was classified as Type III C according to the Gustilo-Anderson system, with no concurrent injuries noted in the systemic assessment except for the affected limb (Fig. 1). The initial vital signs were as follows: blood pressure, 90/60 mmHg; pulse rate 160/min, respiratory rate 40/min, and body temperature, 38°C. Neurological examination showed a Glasgow Coma Scale score of 15, except for a limb injury that caused sensation and movement loss. Admission tests showed leukocytosis (WBC 15.7), 85% neutrophils, and hemoglobin level of 8.5 g/dl. The wound was infected with *Staphylococcus aureus* and Pseudomonas species, requiring immediate antibiotic therapy based on sensitivity.

Interventions were initiated, including the application of a tourniquet, admission to the operating theatre, and administration of general anesthesia. Blood transfusions and rehydration were then performed. In the operating theatre, a comprehensive evaluation confirmed the extensive nature of injuries. Vascular consultation revealed a ruptured posterior tibial artery resulting in the absence of capillary refill. Neurosurgical evaluation revealed loss of sensation and movement, warranting further imaging. Using the Mangled Extremity Severity Score (MESS) and Limb Salvage Index (LSI), the severity of the injury was quantified as 8 and 7, respectively.

**Figure 2:**
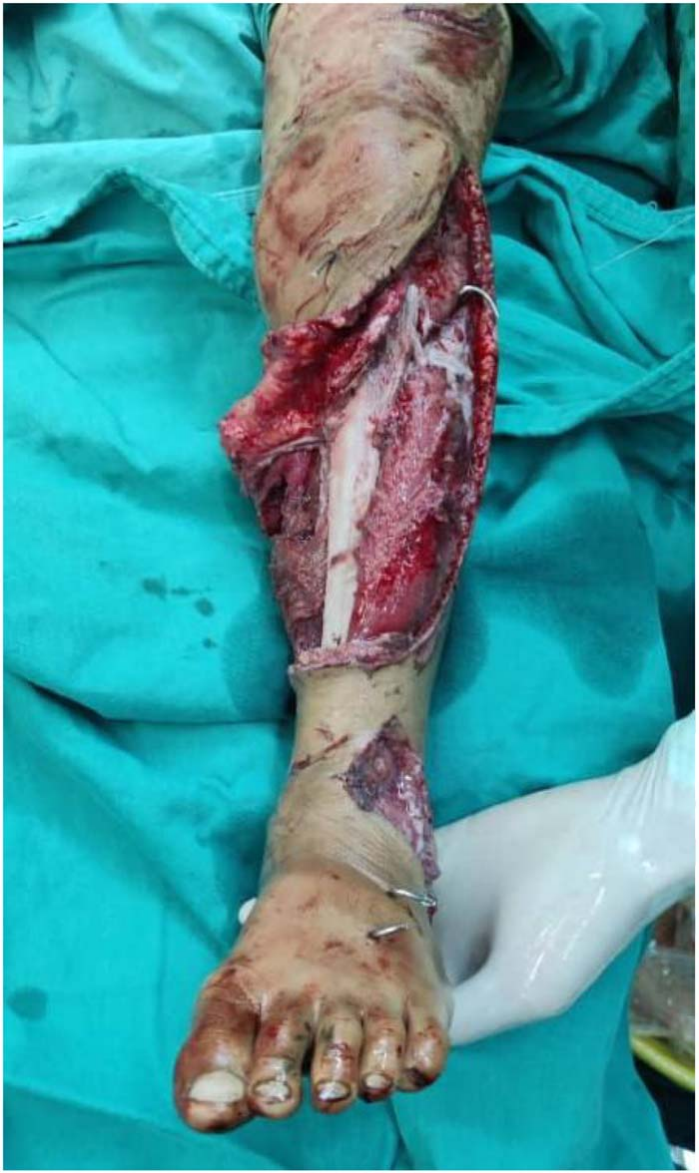
Shows the leg following the placement of the K-wire and prior to suturing the skin flap.

**Figure 3:**
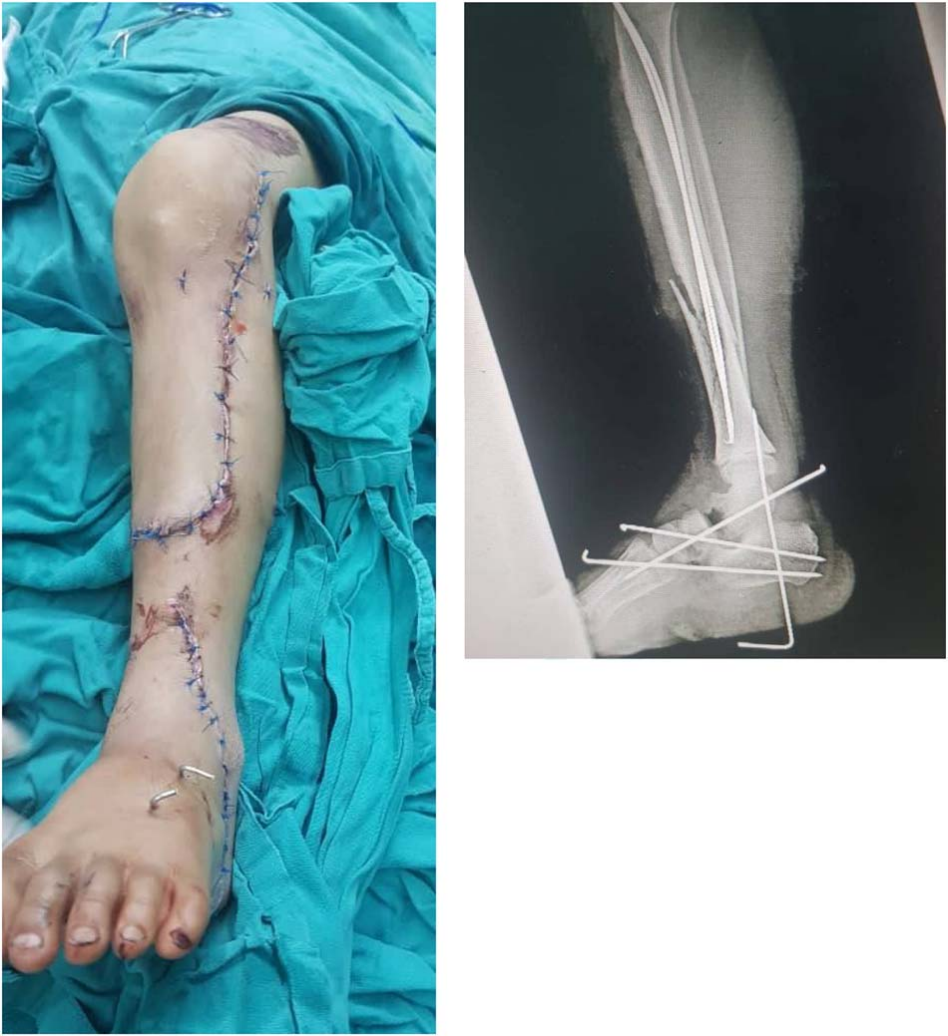
Displays the leg after suturing the skin flap, along with the X-ray image following the installation of the K-wires.

**Figure 4:**
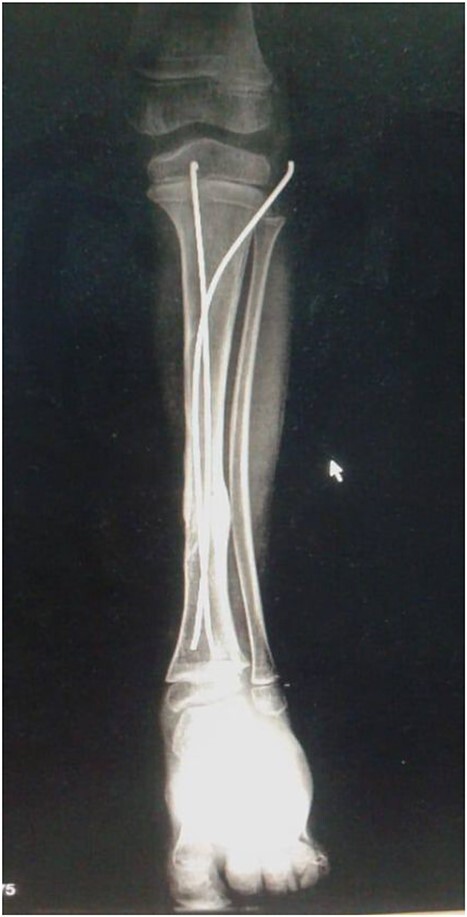
Shows the leg 2 months post-surgery, following the removal of wires.

**Figure 5:**
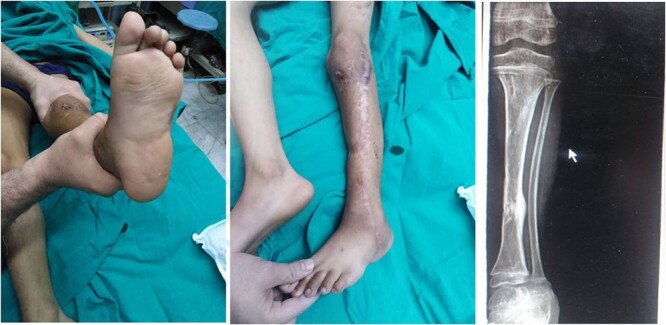
Shows the leg after 8 months post-surgery, indicating successful completion of the recovery process.

The decision to perform limb salvage surgery was made despite parental refusal and the consideration of the patient’s age. Initial debridement was performed in the supine position, with dislocations and fractures reduced and fixed using K-wires (Fig. 2). A temporary skewer was placed between the foot and the leg components to ensure stability. The patellar tendon, posterior muscles, and skin flap were meticulously sutured (Fig. 3). Postoperative follow-up showed satisfactory capillary refill, moderate sensation, and return of the anterior tibial artery pulse within three days. Progressive wound healing was observed over a month, allowing partial weight-bearing ambulation at 2 months (Fig. 4). Complete leg bone healing was achieved at 6 months, with removal of the flexible nail and restoration of the full range of motion and sensation (Fig. 5).

## Discussion

Severely injured extremities often lead to high amputation rates because of the priority given to managing life-threatening injuries over limb trauma. In the acute phase, definitive treatment of severely injured extremities, with the exception of primary amputation, is rarely considered necessary. The definition of a managed extremity varies and can affect multiple systems, resulting in questioned limb viability and limb loss being a probable outcome [7]. The injuries in our patients aligned with the latter definition, making the decision between amputation and salvage challenging, especially in pediatric cases, considering the child’s ongoing growth and development and initial parental reluctance toward amputation.

Advancements in limb salvage techniques have enabled the preservation of limbs that might have previously required amputation [7]. MESS is a widely used scale in orthopedic literature to assess limb injuries and guide decisions on limb salvage [8]. For pediatric patients, MESS adjustments have a threshold of ≥6.5, indicating a high risk of amputation, while the threshold for adults is ≥7 [9]. In this case, the MESS was 8 and LSI was 7. However, commonly used scoring systems lack precision in predicting functional outcomes and deciding between limb salvage and amputation for lower extremity injuries.

Caution should be exercised when relying excessively on microvascular and novel techniques because they can lead to higher mortality, morbidity, hospital stay, and cost [10]. Experienced decision making is crucial. A study on open tibial fractures in children showed that their superior healing capacity, salvaging potential, and bone-forming capability make them different from adults [11]. The sequence of approaches to vascular and skeletal injuries remains debated, with revascularization prioritized over skeletal stabilization when feasible Popliteal Vessel Trauma: Surgical Approaches and the Vessel-First [5]. However, some patients with severe bleeding fractures may benefit from orthopedic stabilization before vascular repair without an increased risk of limb loss [5, 12]. In our case, initial debridement and skeletal stabilization were prioritized because of the short ischemia time.

We used K-wires and a flexible nail for bone stabilization, which is a cost-effective, globally available, and easily removable method that allows early mobilization and prevents joint stiffness [13]. Our hypothesis was supported by a retrospective study showing a lower complication rate and fewer pin-site infections with percutaneous K-wire fixation than with external fixation [14, 15]. The child showed full range of motion 8 months post-surgery, indicating promising recovery. Further studies are needed to establish clear guidelines and optimal treatment strategies for the management of complex fractures in children and adolescents.

## Data Availability

All data were generated during the manuscript and if there any additional data its available upon request.
